# DNAJC12 as a Mediator Between ESR1 and ERBB4 in Breast Carcinoma Cells

**DOI:** 10.3389/fonc.2021.582277

**Published:** 2021-02-24

**Authors:** Mianjie Lin, Ya-Nan Wang, Yixin Ye, Zhelei Xiong, Fengbiao Guo, Haibin Chen

**Affiliations:** ^1^ Guangdong Provincial Key Laboratory of Breast Cancer Diagnosis and Treatment, Shantou University Medical College, Shantou, China; ^2^ Department of Histology and Embryology, Shantou University Medical College, Shantou, China

**Keywords:** ESR1, DNAJC12, ERBB4, breast carcinoma, signaling pathway

## Abstract

Mutation of the DNAJC12 gene is typically associated with non-progressive Parkinsonism, but is also detectable in breast carcinoma where its contribution and mechanisms are unexplored. In breast carcinoma, ESR1 was positively correlated with DNAJC12 and ERBB4, and DNAJC12 was positively correlated with ERBB4. We used the GEO2R tool to compare differential gene expression of MCF-7 cells, following ESR1 knockdown (GEO database, E-GEOD-27473 array), and found decreased expression of DNAJC12 and ERBB4 in ESR1-silenced MCF-7 cells. The number of identical genes having correlativity with ESR1, DNAJC12, or ERBB4 was 12,165 (66.41%). These results suggest that ESR1 can promote the expression of DNAJC12 and ERBB4, and DNAJC12 can enhance the expression of ERBB4 in MCF-7 cells, implying that there may be a regulatory network among these three genes.

## Introduction

Breast carcinoma is one of the most common malignancy and predominant cause of cancer-related death in women around the world, with a one in seven lifetime risk (1–3). Breast cancer, for the majority of patients, is treated with upfront surgery followed by other adjuvant (post-operative) modalities including radiotherapy, chemotherapy, endocrine therapy, immunotherapy, and adjuvant therapy of traditional Chinese medicine (4–9). Estrogen receptor (ER)-positive and/or progesterone receptor (PR)-positive breast cancer accounts for approximately 70% of all breast cancers, and 85% of those in women over 70 years of age (10–12). ER-positive tumors often respond poorly to neoadjuvant chemotherapy and therefore require robust alternatives (13–16). So far, three categories of neoadjuvant endocrine therapy are available: selective ER modulators (primarily tamoxifen), selective ER degraders (fulvestrant), and aromatase inhibitors (including letrozole, anastrozole, and exemestane) which block estrogen synthesis (17). However, the adoption of neoadjuvant endocrine therapy for ER-positive tumors has been much slower and such treatments have serious side effects that can lead to poor quality of life for patients (18). Thus, biological targeted therapy is concerned by more and more medical workers.

Heat shock proteins (HSPs) are a class of heat stress proteins that exist widely from bacteria to mammals, many of which have molecular chaperone activity, and play an important role in the occurrence and development of cancer. HSPs are mainly induced by heat shock or other stresses to aid in the folding, processing, and maturation of auxiliary proteins, and can be classified into Hsp27, Hsp40, Hsp60, Hsp70, Hsp90, and larger HSPs according to molecular mass (19). Several heat shock protein genes are deregulated in all molecular subtypes of breast carcinoma while others appear deregulated in specific molecular subtypes, and the overall survival of breast carcinoma patients appears associated with the expression level of certain HSPs (20). In addition, HSPs play important roles in breast carcinoma, including regulating the status of extracellular matrix proteins (21), maintaining the survival and proliferation of carcinoma cells (22, 23), regulating protein expression and maintaining stability (24, 25), and regulating the cell cycle and migration (26). Many studies have proved that HSPs are beneficial for the diagnosis, treatments, and efficacy evaluation of cancers. HSPs may also have potential clinical uses as biomarkers for cancer diagnosis, for assessing disease progression, or as therapeutic targets for cancer therapy (27–32).

DNAJC12, also known as JDP1 or HPANBH4, is a member of the DnaJ heat shock protein family (Hsp40) C12 (33, 34). The mRNA expression level of DNAJC12 is generally higher in breast carcinoma (35), especially in ESR1-positive breast carcinoma tissues (36). ESR1 is associated with multiple HSPs, such as Hsp27, Hsp70, Hsp90 (37–39), and in ESR1-positive human MCF-7 breast carcinoma cells, 17ß-Estradiol can induce DNAJC12 mRNA expression (36). However, the relationship between ESR1 and DNAJC12 is still unclear, and the signaling pathway is also unknown. In this study, we explored the relation between ESR1 and DNAJC12, and predicted DNAJC12-related genes involved in signaling pathways so as to offer a preliminary theoretical basis for the expression and function of DNAJC12 in breast carcinoma, which may provide a new targeted therapy site of breast cancers.

## Materials and Methods

### Microarray Source

The gene expression dataset (E-GEOD-27473) analyzed in this study was obtained from EMBL (https://www.ebi.ac.uk/), and was comprised of three profiles of MCF-7 cells with normal ESR1 expression and three profiles of MCF-7 cells with the ESR1 gene silenced (40).

### Plasmids

Plasmids encoding ESR1 and DNAJC12 cDNAs, cloned into the pcDNA3.1 + vector, were purchased from the Hangzhou Yanzhen Biotechnology Co., Ltd. and extracted with a FastPure Plasmid Mini Kit (Vazyme Biotech, DC201).

### Analyzing of Gene Chip by GEO2R Tool

GEO2R (https://www.ncbi.nlm.nih.gov/geo/geo2r/) is an interactive web tool that allows users to compare two or more groups of samples in order to identify genes that are differentially expressed. GEO2R tool was used to identify the differentially expressed genes in the dataset (E-GEOD-27473), and the gene expression values and adjusted p-values of genes (ESR1, DNAJC12, and ERBB4) were saved in this study.

### Predicting ESR1 Binding Sites by PROMO

PROMO (41, 42), a virtual laboratory for the study of transcription factor binding sites in DNA sequences, was used to search for the potential binding sites of ESR1 on the promoter region of DNAJC12 in this study.

### Analysis of TCGA data by cBioPortal

The cBioPortal for Cancer Genomics (http://www.cBioPortal.org/) is an online tool providing visualization, analysis, and downloading cancer genomic datasets (43, 44). In this study, 1,108 invasive breast carcinoma samples from the TCGA database were used to identify genes correlated with ESR1, DNAJC12, and ERBB4. According to the key genes of signal pathways provided by the cBioPortal, the VBA program was used to screen the genes having a correlation with DNAJC12 (45). Then, PCR detection was employed to verify differential expression of the identified genes. The R program was used to draw the Venn diagram for analyzing the distribution of those genes whose “q-value” was less than or equal to 0.05 in the lists related to the three genes.

### Cell Culture

MCF-7 cells (ATCC cell bank) used in this study were kindly provided by Professor Wenxiu Ni (Department of Chemistry, Shantou University Medical College). Cells were cultured with high glucose DMEM medium (Gibco, C11995500BT) containing 10% FBS (Gibco, 16010-159) at 37°C with 5% CO_2_. MCF-7 cells were transfected with ESR1 or DNAJC12 plasmids for 0, 24, 48, and 72 h when cells reached 70~90% confluence.

### Extraction of Total Cellular RNA and PCR

Total RNA of cultured cells was extracted using Trizol (TIANGEN, DP405). Subsequent RT-PCR was performed using a HiScriptII 1st Strand cDNA Synthesis Kit (Vazyme Biotech, R212-02). Sequences of primers are listed in **Table 1**. PCR reaction conditions involved a pre-denaturation in 95°C for 3 min, 29 PCR cycles for amplification (each consisting of 15 s of denaturation at 95°C, 30 s of annealing at 56°C, and 30 s of extension at 72°C), 10 min at 72°C for final extension, then stored at 4°C. PCR products were subjected to agarose gel electrophoresis.

**Table 1:** Base Sequence of Primers.

Gene	Primer sequence (5′→3′)
DNAJC12	F: CAGACAAGCATCCTGAAAACCC
	R: TCGCCAGTGGTCATAGCGGGC
APH1B	F: TCTACTGATTTCGTCCCT
	R: TTTATAATATGCAAATCGGAAC
CCNE1	F: TGCCTTGAATTTCCTTATGGTA
	R: AGTTCTCTATGTCGCACCAC
IGF1R	F: CGCCTCCAACTTCGTCT
	R: TCCTCAACTTGTGATCCGTA
BCL2	F: ACTGAGATTTCCACGCCGAAG
	R: TTCTCGGCACAATTGGTAGCTT
ERBB4	F: TTTACGCATTATTCGTGGGA
	R: CAGTACAGGACTTATGGCAAC
β-Actin	F: GAGCTACGAGCTGCCTGACG
	R: GTAGTTTCGTGGATGCCACAG

### Western Blot

MCF-7 cells were harvested, and then lysed in RIPA buffer containing PMSF (Solarbio, R0010) and proteinase inhibitors (Sangon Biotech, C600386-0001), and the lysates were clarified by centrifugation at 12,500 rpm for 15 min at 4°C. A BCA Protein Assay Kit (Beyotime Biotechnology, P0012) was used to determine protein concentration. Total cellular protein was separated by SDS-PAGE and electro-transferred onto PVDF membranes (Millipore, ISEQ00010). Then the membranes were blocked with 5% milk powder (Sangon Biotech, A600669-0250) and washed by 0.1% TBS/T for 10 min. Subsequently, the membranes were incubated with primary antibody. The following primary antibodies were used: mouse monoclonal anti-ESR1 (Santa Cruz Biotechnology, sc-8002), rabbit polyclonal anti-DNAJC12 (Proteintech, 12338-1-AP), rabbit polyclonal anti-ERBB4 (Proteintech, 19943-1-AP), or mouse monoclonal anti-β-actin (Beyotime Biotechnology, AF0003) at 4°C overnight. After washing three times in 0.1% TBS/T, membranes were incubated with the secondary HRP-conjugated goat anti-rabbit IgG (H + L) (Beyotime Biotechnology, A0208) or HRP-conjugated goat anti-mouse IgG (H + L) (Beyotime Biotechnology, A0216). Proteins were visualized with an ECL system.

### Statistical Analysis

Statistical analysis was conducted using Image J software, GraphPad Prism 7, or SPSS software version 20.0. Additionally, when the variances were homogeneous, an LSD test was applied to make pairwise comparisons. The Games-Howell test was applied to make pairwise comparisons when the variances were uneven. Differences between groups were evaluated by one-way ANOVA (*: *P* < 0.05, **: *P* < 0.01, ***: *P* < 0.001, ****: *P* < 0.0001.). Data were expressed as mean ± SEM of at least three independent experiments. A *p*-value < 0.05 was considered to be statistically significant.

## Results

### ESR1 Promotes Expression of DNAJC12

**Figure 1:**
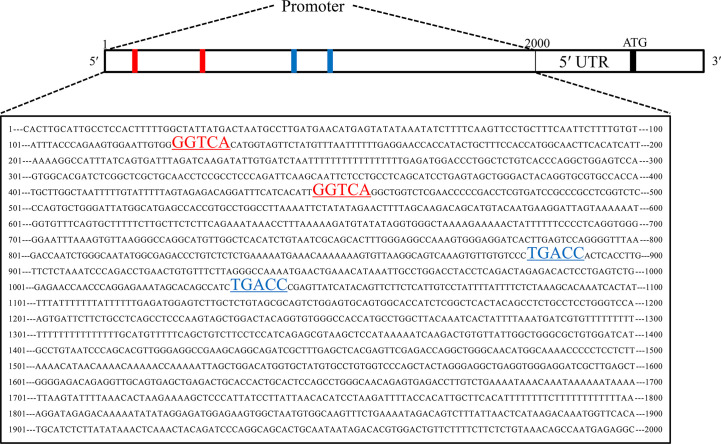
Predicted ESR1 binding sites. The first base to the 2,000^th^ base is DNAJC12 promoter, the red underlined areas and blue underlined areas were both predicted ESR1 binding sites.

**Figure 2:**
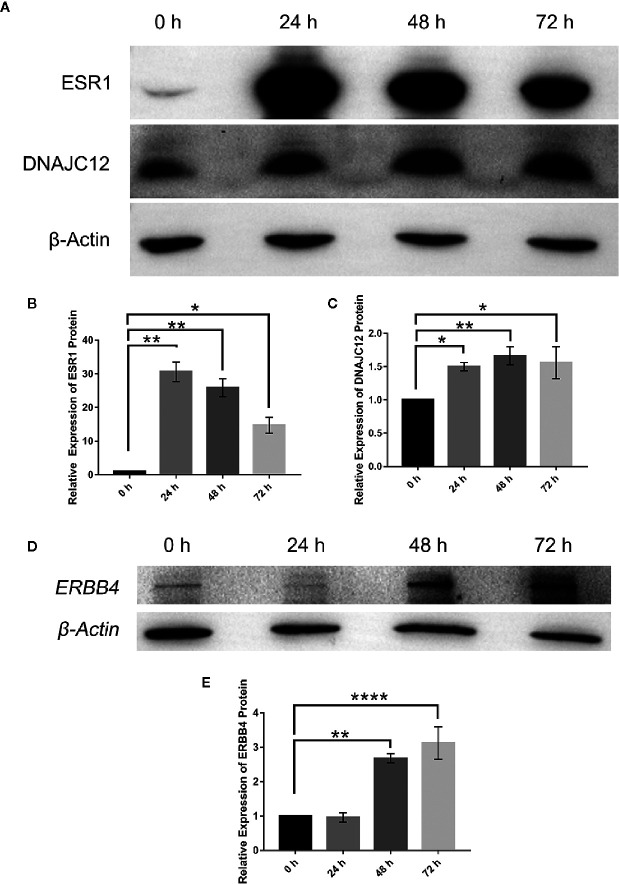
Effect of ESR1 protein overexpression on the expression of DNAJC12 protein and ERBB4 protein. **(A)** MCF-7 cells were transfected with ESR1 plasmids and at 0, 24, 48, and 72 h after transfection, western blotting was performed to detect protein expression of ESR1 and DNAJC12 (n = 5). **(B)** Bar chart of ESR1 protein expression level. **(C)** Bar chart of DNAJC12 protein expression. **(D)** MCF-7 cells were transfected with ESR1 plasmids, and at 0, 24, 48, and 72 h after transfection, western blotting was performed to detect expression of ERBB4 (n = 3). **(E)** Bar chart of ERBB4 protein expression. (*P < 0.05, **P < 0.01, ****P < 0.0001).

To explore the upstream regulatory genes of DNAJC12 in MCF-7 cells through GEO2R, it was shown that the mRNA level of DNAJC12 was reduced in ESR1-silenced MCF-7 cells (**Tables 2** and **3**). Also, ESR1 has four potential binding sites on the DNAJC12 promoter (**Table 4** and **Figure 1**) at the 125^th^ nucleotide, 450^th^ nucleotide, 886^th^ nucleotide, or 1,034^th^ nucleotide. The dissimilarity indices were less than 0.000001, indicating that the predicted binding sites were highly matched. MCF-7 cells were treated with ESR1 plasmid, then examined DNAJC12 expression at various times, and it was found that the expression of DNAJC12 protein was increased (**Figures 2A–C**). These all indicate that ESR1 is an upstream gene of DNAJC12 and can promote the expression of DNAJC12 in MCF-7 cells.

**Table 2:** ESR1 mRNA expression in gene chip (E-GEOD-27473).

Sample	Title	Value
GSM678802	MCF-7, biological rep1	12.2408
GSM678803	MCF-7, biological rep2	12.2049
GSM678804	MCF-7, biological rep3	12.2326
GSM678805	MCF-7 silenced estrogen receptor, biological rep1	4.47218
GSM678806	MCF-7 silenced estrogen receptor, biological rep2	4.38426
GSM678807	MCF-7 silenced estrogen receptor, biological rep3	4.34567

adj.P.Val = 0.00000000000329.

Value: mRNA expression level.

**Table 3:** DNAJC12 mRNA expression in gene chip (E-GEOD-27473).

Sample	Title	Value
GSM678802	MCF-7, biological rep1	8.71679
GSM678803	MCF-7, biological rep2	8.81721
GSM678804	MCF-7, biological rep3	8.74445
GSM678805	MCF-7 silenced estrogen receptor, biological rep1	6.38893
GSM678806	MCF-7 silenced estrogen receptor, biological rep2	5.69465
GSM678807	MCF-7 silenced estrogen receptor, biological rep3	5.78474

adj.P.Val= 0.0000000987.

Value: mRNA expression level.

**Table 4:** Potential ESR1 binding site on the DNAJC12 promoter region.

Factor name	Start position	End position	Dissimilarity	String
ESR1 [T00261]	125	129	less than 0.000001	GGTCA
ESR1 [T00261]	450	454	less than 0.000001	GGTCA
ESR1 [T00261]	886	890	less than 0.000001	TGACC
ESR1 [T00261]	1034	1038	less than 0.000001	TGACC

The base positions in the table are consistent with those in **Figure 1**.

### DNAJC12 Enhances Expression of ERBB4

**Figure 3:**
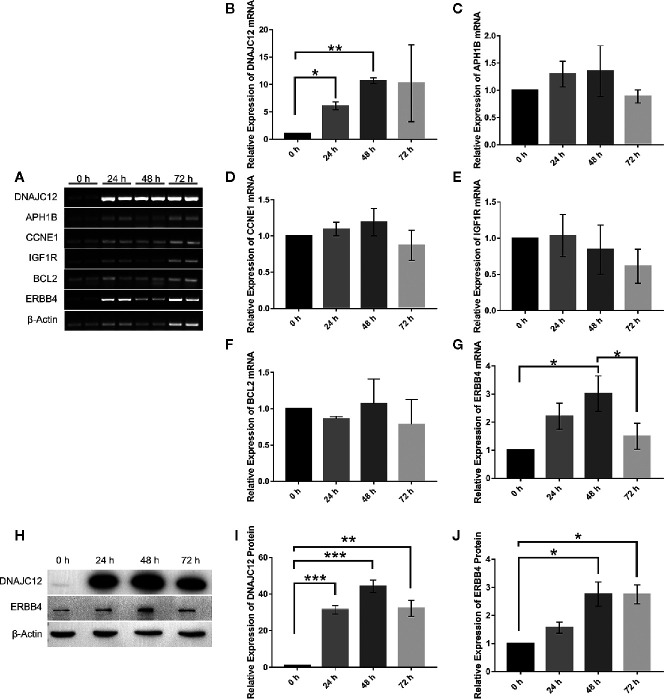
Effect of DNAJC12 overexpression on expression of various genes. **(A)** MCF-7 cells were transfected with DNAJC12 plasmids, and then the mRNA of various genes was detected by PCR at 0, 24, 48, and 72 h (n = 3). **(B)** DNAJC12 mRNA expression. **(C)** APH1B mRNA expression. **(D)** CCNE1 mRNA expression. **(E)** IGF1R mRNA expression. **(F)** BCL2 mRNA expression. **(G)** ERBB4 mRNA expression. **(H)** MCF-7 cells were transfected with DNAJC12 plasmids, and then western blotting was used to detect the protein expression levels of DNAJC12 and ERBB4 at 0, 24, 48, and 72 h (n = 5). **(I)** DNAJC12 protein expression. **(J)** ERBB4 protein expression. (*P < 0.05, **P < 0.01, ***P < 0.001).

With the help of VBA programming language and key genes of the signaling pathways provided by cBioPortal, the candidate genes were obtained, including CCNE1, APH1B, IGF1R, BCL2, and ERBB4 (**Table 5**), which may be related to DNAJC12 in MCF-7 cells. Then, it was found that only ERBB4 was significantly different after detecting the mRNA expression levels of these candidate genes by PCR (**Figures 3A–G**). Moreover, the same trend was observed at the protein level (**Figures 3H–J**). These results indicate that in MCF-7 cells, ERBB4 expression can be elevated by DNAJC12.

**Table 5:** Screening results of gene group having correlativity with DNAJC12 by VBA program.

Correlated Gene	Cytoband	Spearman’s Correlation	*P*-value	Q-value	Involvement in signaling regulation and control
CCNE1	19q12	−0.531	3.84E-81	6.41E-79	Cell cycle control
APH1B	15q22.2	0.583	2.59E-101	1.94E-98	Notch signaling
IGF1R	15q26.3	0.537	4.29E-83	8.94E-81	Other growth/proliferation signaling, RTK signaling family
BCL2	18q21.33	0.603	1.09E-109	1.38E-106	Survival/cell death regulation signaling
ERBB4	2q34	0.550	3.63E-88	1.06E-85	RTK signaling family

### ESR1 Up-regulates Expression of ERBB4

Based on previous results in this study, a new problem was arisen that whether ESR1 was also the upstream gene of ERBB4. ERBB4 may be regulated by ESR1 according to our results showing that ESR1 can elevate the expression of DNAJC12 (**Figures 2A–C**) and DNAJC12 can enhance the expression of ERBB4 **(** **Figure 3**). Therefore, ERBB4 expression was detected by western blotting following transfection of ESR1-overexpressed plasmid in MCF-7 and it was shown that the expression level of ERBB4 was increased (**Figures 2D, E**). Furthermore, re-analysis of E-GEOD-27473 noticed that the ERBB4 mRNA expression was decreased following ESR1 silencing (**Table 6**), which was consistent with our previous results (**Figure 2D**). In brief, ESR1 is an upstream gene of ERBB4 and can up-regulate the expression of ERBB4 in MCF-7 cells.

**Table 6:** ERBB4 mRNA expression in gene chip (E-GEOD-27473).

Sample	Title	Value
GSM678802	MCF-7, biological rep1	7.0086
GSM678803	MCF-7, biological rep2	7.03895
GSM678804	MCF-7, biological rep3	6.90234
GSM678805	MCF-7 silenced estrogen receptor, biological rep1	4.49171
GSM678806	MCF-7 silenced estrogen receptor, biological rep2	4.58529
GSM678807	MCF-7 silenced estrogen receptor, biological rep3	4.37605

adj.P.Val=0.00000000191.

Value: mRNA expression level.

### Positive Correlations Among the Three Genes

**Figure 4:**
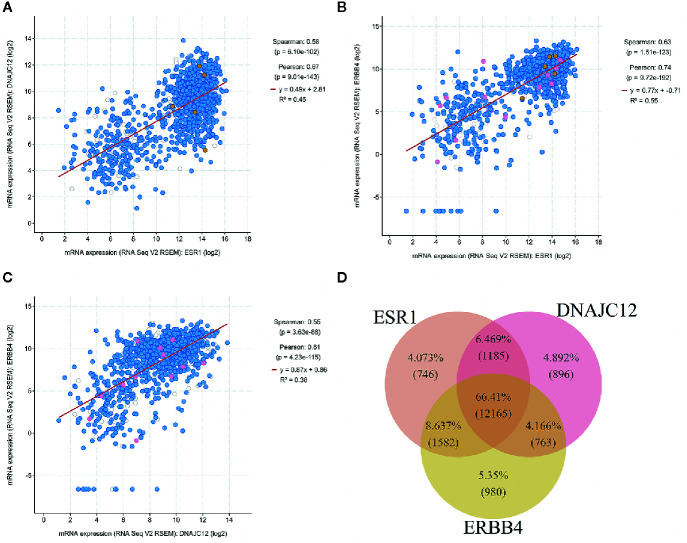
Analysis of breast carcinoma samples from the TCGA database. **(A)** Relational diagram between mRNA expression levels of ESR1 and DNAJC12. **(B)** Relational diagram between mRNA expression levels of ESR1 and ERBB4. **(C)** Relational diagram between mRNA expression levels of DNAJC12 and ERBB4. **(D)** Venn diagram of genes having correlativity with ESR1, DNAJC12, and ERBB4. The flesh color circle represents genes having correlativity with ESR1, the pink circle represents genes having correlativity with DANJC12, and the claybank circle represents the genes having correlativity with ERBB4.

The cBioPortal tool was employed to analyze the TCGA database for correlativity among the expression of ESR1, DNAJC12, and ERBB4 in breast carcinoma. ESR1 mRNA expression was positively correlated with the mRNA expression levels of DNAJC12 and ERBB4, with Spearman rank correlation coefficients of 0.58 and 0.63, respectively (**Figures 4A, B**). In addition, the mRNA expression levels of DNAJC12 and ERBB4 showed a positive correlation, and with a Spearman rank correlation coefficient of 0.55 (**Figure 4C**).

### Analysis Number of Genes Related to ESR1, DNAJC12, and ERBB4

A new problem was arisen whether there are genes in common that correlate with ESR1, DNAJC12, and ERBB4 expression in breast carcinoma, the cBioPortal tool was first used to output the lists containing genes related to ESR1, DNAJC12, or ERBB4. Secondly, using the VBA program, we identified a number of genes from the lists and the genes were stored in three separate lists. Last, the genes from new lists were used to draw a Venn diagram by R language (**Figure 4D**). The number of common genes (i.e. the overlapping parts of the three circles) was 12,165, accounting for 66.41%, while the non-overlapping parts contained a total of 2,622 genes (including 746 genes related to ESR1, 896 genes related to DNAJC12, and 980 genes related to ERBB4), only accounting for 14.315%. In addition, the total number of two circular overlapping parts was 3,530, whose total percentage was 19.272%. Judging from the percentage, the number of identical genes having correlativity with ESR1, DNAJC12, and ERBB4 respectively was 12,165, and its proportion was 66.41%, which accounts for the majority of genes in the three independent lists.

## Discussion

These studies suggest that ESR1 and DNAJC12 may have a certain relationship in breast carcinoma. ERBB4 is a receptor tyrosine kinase that plays a very important role in the development of the mammary gland (46). Studies have shown that this protein expression is related to ESR1 (47–50), but no reports have shown that there is a regulatory relationship between ERBB4 and DNAJC12 protein.

The relation between ESR1 and DNAJC12 was explored in the study. The VBA program and the genes of signal pathways provided by cBioPortal were combined to create an Excel macro workbook to predict DNAJC12-related genes involved in multiple signaling pathways. It was shown that ESR1 and ERBB4 are upstream and downstream genes of DNAJC12, respectively. ESR1 positively correlates with DNAJC12 and ERBB4, and DNAJC12 also positively correlates with ERBB4 in breast carcinoma.

Currently, DNAJC12 has been seldom researched in breast carcinoma, and the relation between DNAJC12 and ESR1 is still unclear. Analysis of gene expression showed that when expression of ESR1 is decreased in MCF-7 cells, DNAJC12 mRNA is also decreased, similar to that found in prior research (36). DNAJC12 promoter region has potential ESR1 binding sites, suggesting DNAJC12 expression could be regulated by ESR1, which is supported by our demonstration that protein expression of DNAJC12 is increased following overexpression of ESR1. All signs point to the conclusion that ESR1 acts upstream of DNAJC12 to enhance DNAJC12 expression in breast carcinoma. However, it should be noted that there is another mechanism for regulating the expression of DNAJC12. In prostate cancer cells, the mRNA expression of DNAJC12 can be also enhanced by AIbZIP (androgen-regulated transcription factor) located in the endoplasmic reticulum, which is relevant to the androgen and endoplasmic reticulum stress (34, 51, 52). Thus, the expression of DNAJC12 may be regulated by more than one protein, and may also be regulated by different proteins in different cell lines.

Overexpression of the carboxyl terminus sequence (CHIP) of Hsc70 in MCF-7 cells can decrease the protein level of endogenous ESR1, and furthermore attenuate ESR1-mediated gene expression (53). Also, in non-stressed LNCaP cells, DNAJC12 generally binds to Hsc70 (34). So, it can be speculated that if the interaction between DNAJC12 and Hsc70 in breast carcinoma cells is the same as that in LNCaP cells, DNAJC12 is bound to Hsc70, and the CHIP structure of Hsc70 is used to inhibit the expression of ESR1 protein, thus inhibiting the gene transcription mediated by ESR1. So far, the regulatory chain between ESR1 and DNAJC12 has been revealed, but the signaling pathways involving by DNAJC12 is still unclear.

In order to explore the signaling pathways associated with DNAJC12, the data from 1,108 breast carcinoma samples in the TCGA database was analyzed by using the cBioPortal tool and VBA program to obtain five candidate genes. Through a series of experiments, we confirmed that ERBB4 is a downstream gene of DNAJC12. Additionally, in order to determine the specific signal pathway involved in ERBB4, the KEGG database (https://www.kegg.jp/kegg/pathway.html) was queried and it was found the signal pathway involved in ERBB4 was ERBB signaling pathway (URL: https://www.kegg.jp/kegg-bin/highlight_pathway?scale=1.0&map=map04012&keyword=ERBB4). Thus, ERBB4 is a downstream gene of DNAJC12, and this gene is involved in the ERBB signaling pathway. However, because we did not perform co-immunoprecipitation experiments, there we do not provide direct evidence to confirm whether the association between DNAJC12 and ERBB4 is direct or indirect. And it maybe contains another mechanism, which need to be explored in the future. Hsp47 can bind to DDR2 (discoidin domain receptor tyrosine kinase 2) to DDR2 stability (24). Similar to DDR2, ERBB4 is also a tyrosine kinase. Thus, we speculate that the relationship between DNAJC12 and ERBB4 is similar that between Hsp47 and DDR2.

This research has shown that ESR1 is upstream of DNAJC12 and ERBB4 is downstream of DNAJC12, which raises the question of whether the expression of ESR1 is related to the expression of ERBB4. To test this relationship, the expression of ERBB4 in ESR1-overexpressed MCF-7 cells was examined and found that the expression level of ERBB4 protein is increased, which suggests a relationship between ERBB4 and ESR1, and explains the decrease in ERBB4 mRNA expression level in ESR1-silenced MCF-7 cells indicated by the gene expression dataset (E-GEOD-27473). Also, a positive association between ESR1 and ERBB4 is consistent with the trend shown in two patient datasets (GSE20194 and GSE23988) (49).

ERBB4 and three other tyrosine kinases, ERBB1 (epidermal growth factor receptor), ERBB2 (erb-b2 receptor tyrosine kinase 2), and ERBB3 (erb-b2 receptor tyrosine kinase 3), together form the ERBB protein family (54). In MCF-7 cells, the cytoplasmic domain 2 (CYT-2) of ERBB4 can enhance gene transcription mediated by ESR1 (48). Additionally, ERBB4 generally includes an extracellular domain, a structure near the membrane area (JM-a, rich in cysteine), a structure near the membrane area b (JM-b, rich in cysteine), transmembrane domain, tyrosine kinase domain, cytoplasmic structure 1 (CYT-1), and cytoplasmic structure 2 (CYT-2). After cleavage by TACE (tumor necrosis factor converting enzyme), the extracellular region of ERBB4 protein is released, and γ-secretase can further act on the transmembrane domain and produce soluble intracellular domain (ICD), including the tyrosine kinase domain, CYT-1, CYT-2, and other structures. The CYT-1 structure of the ICD can target the PI3K protein (55). In addition, the expression of ESR1 and ERBB4 can be inhibited by the same miRNAs (hsa−miR−28−5p, hsa−miR−665, and hsa−miR−708−5p), and thus, there may be a competitive combination that may lead to a dynamic balance between the expression of ESR1 and ERBB4 (47). MicroRNAs, including miR-375, miR-592, and miR-135a, can jointly inhibit the expression of ESR1 and ERBB4 (50). In short, ESR1 can promote the expression of ERBB4, and proteases can cleave ERBB4 to generate additional structures. And then, the structures can enhance gene transcription mediated by ESR1, and the expression levels of both ESR1 and ERBB4 may also be constrained by multiple miRNAs.

In addition, in order to further investigate the relationship among the expression of these three genes in breast carcinoma, the cBioPortal tool was employed to analyze the gene expression profile data in the TCGA database. In the result, ESR1 is positively correlated with DNAJC12 and ERBB4, and DNAJC12 is positively correlated with ERBB4. The trend of data distribution is similar to the trend of our cell experimental results. Based on the above analysis, ESR1, DNAJC12, and ERBB4 are found to be closely related. According to the Venn diagram, those genes which were related to these three genes occupied the majority of genes, accounting for 66.41%. This suggests that not only are the three genes related to each other, but also there are many identical genes potentially related to all three genes in breast carcinoma, which imply that there may be a regulatory network among them.

**Figure 5:**
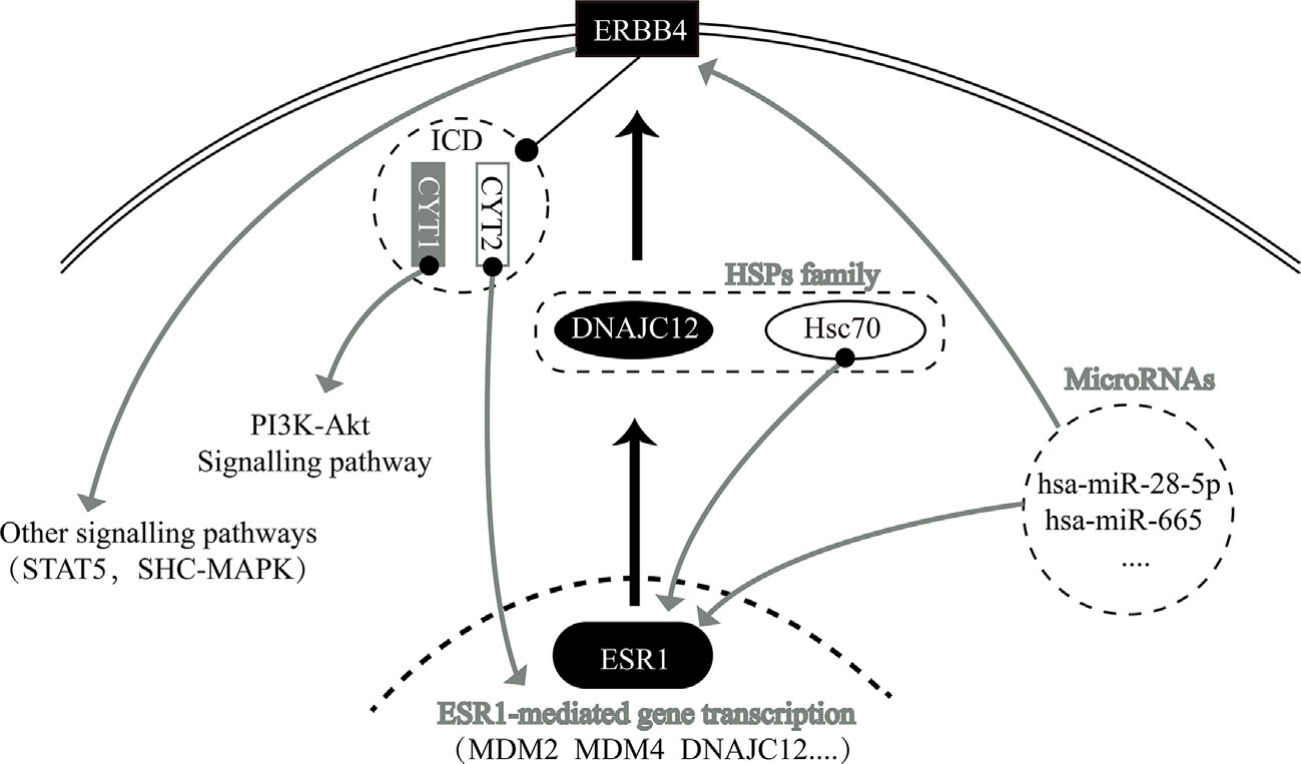
Relationship network diagram among some genes, including ESR1, DNAJC12, and ERBB4.

In summary, we have determined that ESR1 and ERBB4 are upstream and downstream genes, respectively, of DNAJC12 in breast carcinoma. Moreover, ESR1, DNAJC12 and ERBB4 have a regulatory relationship with each other and with other genes (**Figure 5**). Importantly, we demonstrate the status of DNAJC12 in the ERBB signaling pathway, and also provide a new regulatory mechanism between ESR1 and ERBB4.

## Conclusions

Our study shows that ESR1 and ERBB4 are the upstream and downstream genes, respectively, of DNAJC12, making ERBB4 a downstream gene of ESR1. ESR1 is positively correlated with DNAJC12 and ERBB4, and DNAJC12 is positively correlated with ERBB4 in breast carcinoma. The number of genes having correlativity with ESR1, DNAJC12, and ERBB4 is 12,165, accounting for 66.41% of the total number of genes associated with each gene individually, implicating that there may be a regulatory network among ESR1, DNAJC12, and ERBB4.

## Data Availability Statement

Publicly available data sets were analyzed in this study. These data can be found here: EMBL (https://www.ebi.ac.uk/) (E-GEOD-27473) and cBioPortal (http://www.cBioPortal.org/).

## Author Contributions

HC and ML designed the study together, and all members performed all experiments and interpreted the results. ML, YW, and HC wrote the manuscript. All authors contributed to the article and approved the submitted version.

## Funding

This work was supported by the Postgraduate Training Fund of Shantou University Medical College (510817044) and Guangdong Provincial Key Laboratory for Breast Cancer Diagnosis and Treatment (2017B030314116).

## Conflict of Interest

The authors declare that the research was conducted in the absence of any commercial or financial relationships that could be construed as a potential conflict of interest.
